# Promoting Reproducible Research for Characterizing Nonmedical Use of Medications Through Data Annotation: Description of a Twitter Corpus and Guidelines

**DOI:** 10.2196/15861

**Published:** 2020-02-26

**Authors:** Karen O'Connor, Abeed Sarker, Jeanmarie Perrone, Graciela Gonzalez Hernandez

**Affiliations:** 1 Department of Biostatistics, Epidemiology and Informatics Perelman School of Medicine University of Pennsylvania Philadelphia, PA United States; 2 Department of Biomedical Informatics School of Medicine Emory University Atlanta, GA United States; 3 Department of Emergency Medicine Perelman School of Medicine University of Pennsylvania Philadelphia, PA United States

**Keywords:** prescription drug misuse, social media, substance abuse detection, natural language processing, machine learning, infodemiology, infoveillance

## Abstract

**Background:**

Social media data are being increasingly used for population-level health research because it provides near real-time access to large volumes of consumer-generated data. Recently, a number of studies have explored the possibility of using social media data, such as from Twitter, for monitoring prescription medication abuse. However, there is a paucity of annotated data or guidelines for data characterization that discuss how information related to abuse-prone medications is presented on Twitter.

**Objective:**

This study discusses the creation of an annotated corpus suitable for training supervised classification algorithms for the automatic classification of medication abuse–related chatter. The annotation strategies used for improving interannotator agreement (IAA), a detailed annotation guideline, and machine learning experiments that illustrate the utility of the annotated corpus are also described.

**Methods:**

We employed an iterative annotation strategy, with interannotator discussions held and updates made to the annotation guidelines at each iteration to improve IAA for the manual annotation task. Using the grounded theory approach, we first characterized tweets into fine-grained categories and then grouped them into 4 broad classes—*abuse or misuse, personal consumption, mention,* and *unrelated*. After the completion of manual annotations, we experimented with several machine learning algorithms to illustrate the utility of the corpus and generate baseline performance metrics for automatic classification on these data.

**Results:**

Our final annotated set consisted of 16,443 tweets mentioning at least 20 abuse-prone medications including opioids, benzodiazepines, atypical antipsychotics, central nervous system stimulants, and gamma-aminobutyric acid analogs. Our final overall IAA was 0.86 (Cohen kappa), which represents high agreement. The manual annotation process revealed the variety of ways in which prescription medication misuse or abuse is discussed on Twitter, including expressions indicating coingestion, nonmedical use, nonstandard route of intake, and consumption above the prescribed doses. Among machine learning classifiers, support vector machines obtained the highest automatic classification accuracy of 73.00% (95% CI 71.4-74.5) over the test set (n=3271).

**Conclusions:**

Our manual analysis and annotations of a large number of tweets have revealed types of information posted on Twitter about a set of abuse-prone prescription medications and their distributions. In the interests of reproducible and community-driven research, we have made our detailed annotation guidelines and the training data for the classification experiments publicly available, and the test data will be used in future shared tasks.

## Introduction

### Background

Social media has provided a platform for internet users to share experiences and opinions, and the abundance of data available has turned social networking websites into valuable resources for research. Social media chatter encapsulates knowledge regarding diverse topics such as politics [1], sports [2], and health [3]. A 2015 report by the Pew Research Center [4] suggested that 37% of adults online in the United States considered health to be one of the most interesting topics. Users seek and share health-related information on social media regularly, resulting in the continuous generation of knowledge regarding health conditions, drugs, interventions, and health care policies. Social media has become an important source of data, particularly for public health monitoring because the data generated can be collected and processed in near real-time to make population-level estimates. Consequently, social media data have been used for conducting health-related studies such as tracking the spread of contagious diseases such as influenza [5], predicting depression [6], understanding and characterizing people’s health-related choices such as diet [7], and discovering the potential adverse or beneficial effects of medications [8].

Although the volume of data in social media is attractive, owing to the various complexities associated with the data, such as the use of nonstandard language and the presence of misspellings, advanced natural language processing (NLP) pipelines are required for automated knowledge discovery from this resource. These pipelines typically require the application of machine learning approaches, supervised or unsupervised, for information classification and extraction. Unsupervised approaches such as topic modeling are capable of automatically identifying themes associated with health topics from large unlabeled datasets [9]. However, as targeted applications of social media data are being explored, supervised methods are becoming increasingly popular. Supervised machine learning methods are generally more accurate than unsupervised approaches for targeted tasks (eg, adverse drug reaction detection [10] and user sentiment classification [11]), but they require the manual annotation of large datasets. Over the recent years, public releases of manually annotated datasets have significantly contributed to community-driven development of data-centric solutions to important research problems lying at the intersection of data science and health, and these community efforts have been instrumental in progressing toward the benchmarks for these tasks [12].

The importance of building high-quality datasets and annotation processes cannot be overstated—the reliability of the systems and their performance estimates depend directly on it. When annotating datasets for training machine learning algorithms, the standard approach is to have multiple annotators annotate the same sample of data and then compute agreement among the different annotators. Interannotator agreement (IAA) measures provide estimates about how well defined a task is, its level of difficulty, and the ceiling for the performance of automated approaches (ie, it is assumed to be impossible for an automated system to be better than human agreement). IAA values reported for social media–based annotation tasks are often relatively low [13] compared with other data sources because information in social media can be presented in unique ways, often without sufficient context (eg, due to length limitations, as in the case of Twitter). Although significant attention of the informatics research community is directed toward improving machine learning performance numbers—such as F-measure, recall, precision, and accuracy—on standardized datasets, relatively less attention has been paid to improve the qualities of the datasets that are standardized. On the basis of our significant past experience in social media–based NLP and machine learning research, we have established some best practices for preparing health-related research datasets.

### Guidelines and Corpus Development

One of the most important steps in preparing high-quality corpora is the development of detailed and consistent annotation guidelines that are followed by all the annotators involved. Methodically prepared annotation guidelines for a target task have multiple advantages, as outlined below:

1. They enable the annotation process to be more consistent, leaving fewer decisions to the subjective judgments of different annotators. Consequently, this also inevitably improves IAA, naturally raising the performance ceilings for automated systems.
2. Well-defined guidelines document the clinical or public health purposes of the studies, enabling researchers from informatics or computer science domains to better understand the high-level objectives of the studies, thereby helping bridge the gap between the domains.
3. Data science approaches to health-related problems are seeing incremental development (ie, as one problem is addressed successfully, additional follow-up problems are addressed). Therefore, well-defined annotation guidelines can be crucial to enable extensions of the annotated corpora for future studies.
4. Datasets for a specific problem (eg, adverse drug event detection [10,14,15]) are often developed by distinct teams and can be in different languages. If detailed annotation guidelines are prepared and published for each problem, with sufficient explanation behind the decisions made by the annotating team, the guidelines can be used by different research groups. This could facilitate the use of combined datasets and allow systems trained on one dataset to be ported to the others.
5. The considerations documented within the annotation guidelines of one study can be beneficial for research teams developing corpora for other tasks, as they can follow identical standards or make similar considerations.

In addition to datasets and automated systems that are valuable for the health informatics research community, detailed explanations of methods and justifications for annotation guidelines can impact data-centric automation—particularly for domain-specific problems, where the potential for automation is at the exploratory or early development phase.

In this paper, we discuss the preparation of a dataset from Twitter involving misuse- and abuse-prone prescription medications. Prescription medication misuse and abuse, and more generally, drug abuse, is currently a major epidemic globally, and the problem has received significant attention particularly in the United States in recent years because of the opioid crisis. Given the enormity of the problem and the obstacles associated with the active monitoring of drug abuse, recent publications have suggested the possibility of using innovative sources for close-to-real-time monitoring of the crisis [16], particularly social media, where prescription medications, their use, and misuse are publicly discussed [17,18].

### Prescription Medication Abuse and Social Media

The contribution of prescription medications in the broader drug abuse crisis has been well documented and understood over the recent years. Nonmedical use of prescription medications may result in an array of adverse effects, from nonserious ones such as vomiting to addiction and even death. A significant portion of emergency department visits are due to nonmedical use of prescription medications [19]. Distinct classes of prescription medications are misused or abused with differing intents—stimulants such as Adderall, for example, are often used for performance enhancement, whereas opioids, depressants, and benzodiazepines are typically used for the sensations they produce [20]. A 2016 report focusing on the threat of drug abuse published by the Drug Enforcement Agency suggested that the number of deaths involving prescription medications has overtaken those from illicit drugs such as cocaine and heroin combined, for every year since 2002 [21]. The report also stated that approximately 52 people die each day in the United States from prescription medication overdose—a number that has only increased since the publication of the report. A report by the Centers for Disease Control and Prevention showed that of over 40,000 drug overdose deaths in 2013, more than 20,000 were due to prescription drugs [22]. Understandably, the misuse of certain prescription medications, such as opioids, has resulted in more dire consequences than others. Statistics from the WONDER database [23] suggest that the increasing sales in prescription opioids correlate with the steady increase in opioid overdose deaths over 15 years. Unfortunately, because of the absence of effective, timely surveillance approaches, the problem posed by prescription opioids was not fully understood before it reached the level of a national crisis. Recent advances in NLP, social media mining, and, broadly, data science present us with the opportunity of using public social media data as a complementary resource for monitoring and studying prescription medication use and abuse.

In this paper, we do not distinguish between prescription drug misuse and abuse and use these terms interchangeably to represent all types of nonmedical use. There are, however, subtle differences between the definitions of the terms. *Misuse* is defined by the National Institutes of Health (NIH) National Institute on Drug Abuse (NIDA) as a form of nonmedical use that involves “taking a medication in a manner or dose other than prescribed; taking someone else’s prescriptions, even if for a legitimate medical complaint such as pain”; whereas abuse is defined as “taking a medication to feel euphoria (ie, to get high)” [20]. Although misuse is the contrary or improper use of prescribed drugs, which maybe intentional or unintentional, abuse is intentional use for nonmedical purposes [24]. When it comes to the misuse and abuse of prescription medications, as opposed to illicit drugs, social media may provide unprecedented insights because the population-level extent and mechanisms of abuse for different prescription drugs are not known *a priori.* Our overarching focus is to create a Twitter dataset that enables the training of supervised systems to automatically characterize medication abuse–related chatter for large-scale analysis. Publicly available discussions regarding prescription medication abuse may enable us to discover emerging abuse-prone medications, novel methods of abuse, and other related information. Although some data-centric approaches have been published in recent times for leveraging social media data for monitoring prescription medication abuse, there is a lack of (1) clear descriptions of how abuse information is presented in public social media (eg, Twitter), (2) annotated datasets usable for automatic characterization of social media chatter associated with abuse-prone medications, and (3) thorough annotation guidelines that may serve as the groundwork for long-term future research on this topic.

We present here an analysis of how prescription medication abuse information is presented on Twitter, the details of a large-scale annotation process that we have conducted, annotation guidelines that may be used for future annotation efforts, and a large annotated dataset involving various abuse-prone medications that we envision will drive community-driven data science and NLP research on the topic. Although we primarily focus on the annotation process, guidelines, and the data, we also illustrate the utility of the corpus by presenting the performances of several supervised classification approaches, which will serve as strong baselines for future research.

## Methods

### Data Selection and Collection

In consultation with the toxicology expert of our study (JP), we selected 20 medications (generic) to include in the study. We selected drugs belonging to the classes of prescription medications that have been identified as more commonly abused: opioids (including those used for medication-assisted treatment), benzodiazepines, atypical antipsychotics, central nervous system stimulants, and gamma-aminobutyric acid analogs. Table 1 shows the drug categories, generic names, and brand names for the drugs included in this study. All data were collected from Twitter through the public streaming application programming interface (API). The Twitter API allows data collection in real time through the use of keywords. We used the brand and generic names as keywords, as well as common spelling variants for these keywords generated automatically through a data-centric misspelling generator [25]. We only kept tweets that were in English as per the metadata that was available with them during collection. Starting with a large random sample from the entire collected dataset, we applied further filtering to generate a manageable sample for manual annotation. The tweets were filtered by removing retweets and short tweets only with links. After the collection, a sample of the data was selected for preliminary manual inspection. This inspection involved simply reading a set of tweets to (1) ensure that all medications of interest were included, (2) identify which medications occurred too many times, and (3) check if any noisy nondrug keywords had been introduced during the misspelling generation process leading to the collection of large volumes of irrelevant data. During the sampling and analysis, we discovered that stimulants were particularly overrepresented in social media chatter (eg, *Adderall* was mentioned almost as frequently as stopwords such as *the*, *of*, and in the collected dataset). So, we undersampled tweets mentioning stimulants for the final annotation set using random selection without replacement. This set was then passed to the annotators for guideline development and annotation.

The protocol for this study was reviewed by the University of Pennsylvania’s institutional review board and was determined to meet the criteria for exempt human subjects research as all data collected and used are publicly available. In the examples presented in this paper, all identifiers have been removed, and slight modifications have been made to tweets to protect the anonymity of users.

**Table 1:** Main drug categories, generic names, and brand names for prescription medications included in this study.

Drug category	Generic name	Brand name(s)
Opioids	Oxycodone	Oxycontin, Percocet
	Methadone	Dolophine
	Morphine	Avinza
	Tramadol	Conzip
	Hydrocodone	Vicodin, Zohydro
	Buprenorphine	Suboxone
Benzodiazepines	Diazepam	Valium
	Alprazolam	Xanax
	Clonazepam	Klonopin
	Lorazepam	Ativan
Atypical antipsychotics	Olanzapine	Zyprexa
	Risperidone	Risperdal
	Aripiprazole	Abilify
	Asenapine	Saphris
	Quetiapine	Seroquel
Central nervous system stimulants	Amphetamine mixed salts	Adderall
	Lisdexamfetamine	Vyvanse
	Methylphenidate	Ritalin
GABA^a^ analogs	Gabapentin	Neurontin
	Pregabalin	Lyrica

^a^GABA: gamma-aminobutyric acid.

### Guidelines and Annotation

In a preliminary study that paved the way for a long-term project [26], we performed binary annotation of potential medication abuse tweets. In that study, we classified 6400 tweets from 3 abuse-prone medications and 1 non–abuse-prone medication (control medication) as either *abuse indicating* or *non-abuse indicating* for use in the training and testing of automatic classifiers. The guidelines from that study served as the foundation for this study. In addition, the familiarity we gained from that study regarding the information available in the discussions of potential prescription drug abuse informed our decision to expand the number of categories for this classification task. Our annotation entailed labeling tweets into 1 of 4 categories: *potential abuse or misuse*, *non-abuse consumption*, *drug mention only*, and *unrelated*. The annotators were given the following definitions, with examples, to assist in determining the classification of the tweets:

1. Potential Abuse or Misuse (A): These tweets contain possible indications that the user is abusing or is seeking to abuse or misuse the medication. The user may have a valid prescription for the medication, but their manner of use is indicative of abuse or misuse, or the medication may have been obtained illegally. We also include in this category tweets that can possibly indicate abuse without confirming evidence. As the end goals of this project are to identify all potential mentions of nonmedical or improper drug use by users, we do not differentiate between misuse and abuse.
2. Non-abuse Consumption (C): These tweets indicate that the user has a valid prescription for the medication and is taking the medication as prescribed, or is seeking to obtain the medication for a valid indicated reason. Tweets should be placed in this category when there is evidence of possible consumption, but there is no evidence of abuse or misuse. This category only applies to personal consumption.
3. Drug Mention Only (M): In these tweets, the mention of the medication name is not related to wanting, needing, or using the medication either as prescribed or misuse or abuse. For example, these tweets may be sharing information or news about the medication, jokes, movie or book titles, or lines from movies or songs. This category also includes mentions of use by a third person that do not indicate abuse or misuse by that person.
4. Unrelated (U): These tweets mention the medication keywords, but they do not represent the drug and refer to something else.

We decided on these categories and built our initial guidelines using the grounded theory approach [27] whereby each tweet was categorized in terms of the topic of its content, which were eventually mapped to one of the above categories. We trained 4 annotators using the developed guidelines for the manual categorization of the tweets; 2 of the annotators were the primary authors of the guidelines (AU1 and AU2) and the remaining 2 were expert annotators with past experience in similar annotation tasks (AN1 and AN2). The annotation task was started as an iterative process both for training purposes and to test the efficacy and clarity of the guidelines over a small initial dataset. The annotators were instructed to code each tweet into only one category and were asked to create brief notes stating their thought process for instances in which coding was difficult or where they felt that the reason for their decision was not obvious. The notes were used to assist in adjudication and for error analysis, and they helped to highlight areas in which the guidelines were not clear. We executed a total of 4 such iterations over the same dataset, refining the guidelines at each iteration and expanding them to make distinctions between the different categories more explicit.

From the initial topic categorization of the tweets, we added identifying markers that could be found within the tweets to help determine their classifications. With the exception of *unrelated*, these markers were, in effect, all the subcategories identified during annotator training and manual review of the ways users may express use, potential abuse or misuse, consumption, or just the mention of a medication.

For example, an identifying marker of abuse or misuse is the explicit or implied mention of consuming a higher dose of medication than prescribed:

let's see how fast a double dose of hydrocodone will knock me out

thewaitinggame

An identifying marker of consumption is the taking of a prescribed medication as indicated with no evidence of it being abused or misused:

I was prescribed Ritalin by my doctor to help me. i feel more hyper than focused

Meanwhile, a tweet categorized as mention gives no indication that the person mentioning the medication is taking the medication themselves:

the adderall tweets are not even funny to me. if you saw what i see daily at work it wouldn't be funny to you either.

Textbox 1 presents some examples of the descriptions of the identified subcategories, or markers, within each of the broader categories, or classes, detailing the various ways in which abuse-indicating and other information are shared on Twitter. Although we did not code the tweets’ subcategories during annotation, their descriptions and examples were provided in the annotation guidelines, which helped the annotators to be consistent in their decisions. Consequently, the thorough breakdown of these subcategories, or markers, improved agreement between the different annotators. The full annotation guidelines used by the annotators, with details and examples of each subcategory within the 4 classes, are made available with this publication (Multimedia Appendix 1).

**Figure 1:**
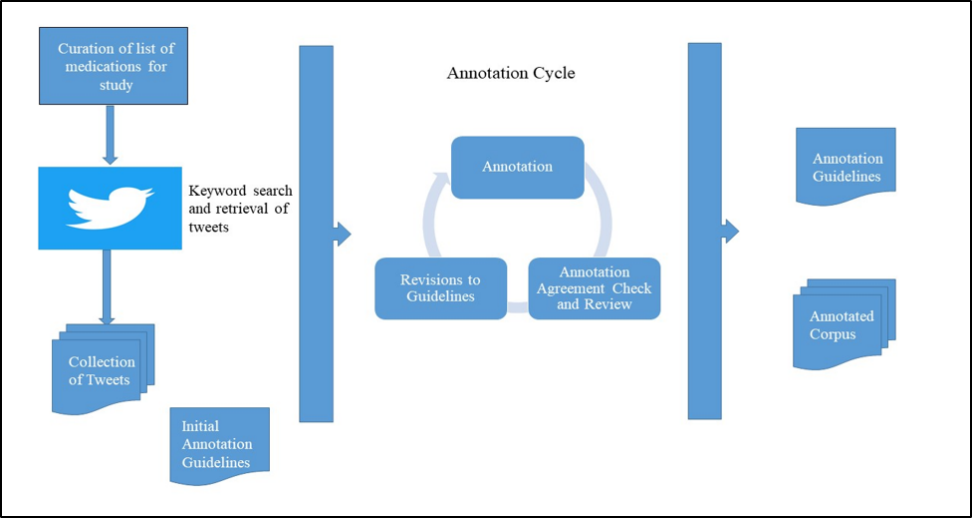
Overview of the creation of the annotation guideline and the iterative annotation process.

The creation of the gold standard corpus commenced after consistent levels of agreement between the annotators were achieved. The corpus of tweets was divided into 3 overlapping sets ensuring that each tweet was annotated at least twice, with some being annotated 3 times. The annotations were completed by 3 expert annotators trained on the guidelines (AU1, AN1, and AN2). The annotators coded each tweet according to the entire text contained in the tweet by following the guidelines established to distinguish between classes. There were no further annotations at the subtweet level. The disagreements from each set were annotated by a fourth annotator (AU2) for resolution. For the tweets that were annotated by 3 annotators, majority agreement was used to resolve disagreements. In the event that all 3 annotators disagreed on the classification, they were reviewed and resolved by AU2. An overview of the process is shown in Figure 1.

**Examples of the descriptions of subcategories or identifying markers for each category from the classification guidelines.**

**1. Potential Abuse or Misuse (A)**

- The tweet explicitly states that the user has taken or is going to take the medication to *experience certain feelings* (ie, to get high) or that the user *experienced certain feelings in the past*.
- The tweet expresses that the user *has or is going to coingest a medication with other prescription medications or illicit drugs or alcohol or coffee* (or other substances).
- The tweet expresses a *mechanism of intake* that is typically associated with abuse or misuse.

**2. Non-abuse Consumption (C)**

- The user mentions *side effects of the drug*, but *there is no implication that these are the result of misusing or abusing the drug*.
- In the tweet, the user expresses *a want for the medication for a condition that matches its indicated use*.

**3. Drug Mention Only (M)**

- The tweet conveys some information about the medication but *contains no indication that the user is taking or wants to take the medication*.
- The mention of the medication is *from a song, book or movie, or some other cultural reference*.
- The mention of the medication *is being used in a joking or a hypothetical statement*.

**4. Unrelated (U)**

- The only tweets that belong to this category are those that include a drug/medication name as keyword, but the keyword is referring to something else and not the drug/medication. It can be, for example, a person’s name or a misspelling of something else.

### Automatic Classification

To demonstrate the utility of the corpus for training systems for automatic classification of medication abuse–related Twitter chatter, we performed a set of supervised classification tasks. Our intent with these experiments was to illustrate that machine learning algorithms are trainable using this dataset and establish a set of baseline performance metrics that can be used as reference for future research. We split the annotated dataset into 2 at approximately 80:20 ratio and used the larger set (13,172/16,443, 80.11%) for training and the smaller set (3271/16,443, 19.89%) for evaluation.

We experimented with 4 classifiers—multinomial naive Bayes (NB), random forest (RF), support vector machines (SVM), and deep convolutional neural network (dCNN). Our extensive past work on social media mining for health research and social media text classification has demonstrated that identifying the best classification strategy requires elaborate experimentation and is best identified by means of community-driven efforts such as shared tasks [12]. Therefore, for the purposes of this study, we did not attempt to identify the optimal classification strategy or perform elaborate feature engineering. Instead, we optimized the specific classifier parameters using 10-fold cross validation over the training sets and only used basic features. For the first 3 classifiers, we used word n-grams (n=1-3) and word clusters [26] as features following basic preprocessing of the texts (lowercasing and stemming). For the dCNN classifier, we used a 3-layer network, and we further split the training set into approximately 80-20 splits and used the larger set for training and the smaller set for validation. We used pregenerated dense word vectors (embeddings) [28] for representing the tweets. All experiments were performed using Python sci-kit learn [29] (NB, RF, and SVM classifiers) and Google’s TensorFlow [30] (dCNN), and the results are presented in the next section.

## Results

### Guidelines and Annotation

**Figure 2:**
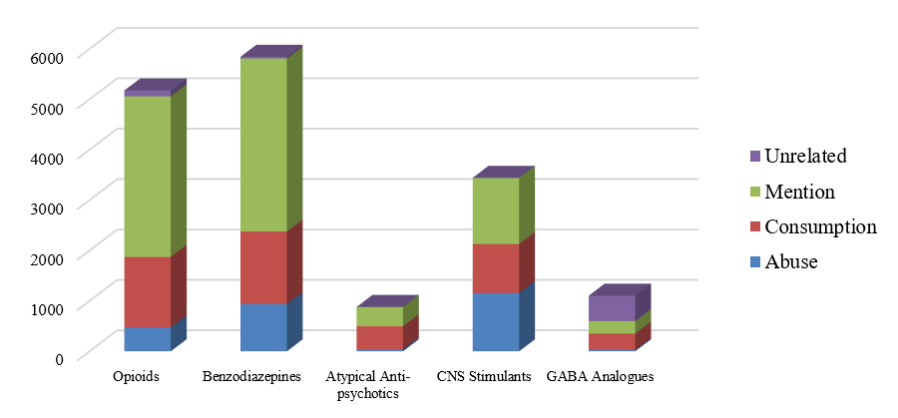
Distribution of tweets in the annotated corpus by annotation category and drug class.

In total, a sample of 16,443 tweets were selected for annotation from more than 1 million posts collected from April 2013 to July 2018. This rather arbitrary number of tweets resulted from the various filtering methods (eg, removing short tweets and undersampling tweets with stimulants) that we applied on a much larger random sample of about 50,000 tweets. Before undersampling, approximately three-quarters of the retrieved tweets mentioned stimulants, and only approximately one-fifth of them were kept following the sampling process. From this chosen set, 517 randomly selected tweets were used in the initial iterations for improving agreement and developing the guidelines. These were then adjudicated and added to the gold standard corpus. The rest of the corpus was split into 3 sets containing 15,405 (set 1), 8016 (set 2), and 6906 tweets (set 3). In addition, a fourth set contained overlapping tweets that were annotated by all 3 of the annotators (set 4). All these sets had an arbitrary number of overlapping tweets with at least one other set, which the annotators were not aware of during annotation. Pairwise IAA, measured using Cohen kappa [31], ranged from 0.681 to 0.971. For the set of tweets with more than two annotators, IAA was measured using Fleiss kappa [32] and was 0.904. IAA for the different sets are reported in Table 2. The final distribution of classes in the corpus, following the completion of the entire annotation process, was 2636 misuse or abuse (16.03%, 2133 in the training set, 503 in the evaluation set), 4587 consumption (27.90%, 3668 in the training set, 919 in the evaluation set), 8565 mention only (52.09%, 6843 in the training set, 1722 in the evaluation set), and 655 unrelated (3.98%, 528 in the training set, 127 in the evaluation set). Figure 2 shows the distribution of tweets and the classes per medication category in the entire collection. The training set tweet texts, along with other resources, will be made available with the final version of this paper [33]. Note that to preserve anonymity of the original posters of the tweets, we will add an additional layer of masking by reposting the tweet texts from our own Twitter profile and sharing the IDs of the tweets posted by this account, along with a download script (written in python). In addition to keeping the original posters anonymous, this method of data sharing will ensure long-term availability of the tweets. We will preserve the test/evaluation set for use in community-driven efforts such as shared tasks.

An analysis of the disagreements suggested that they were somewhat evenly distributed across the categories of interest. Over the first 3 sets, there were a total of 3631 disagreements among the annotators, 1082 (29.80%) were disagreements between abuse or mention classifications, 1160 (31.95%) were between abuse or consumption, 1186 (32.66%) were between consumption or mention, and the remaining 203 (5.59%) were disagreements between unrelated or all other categories. The analyses also showed that the disagreements did not result from the annotators’ incorrect interpretations of the guidelines but from their interpretations of the tweets. We, therefore, concluded that it was unlikely that we could further increase the IAA by updating or modifying the annotation guidelines.

**Table 2:** Annotation agreement results.

Set	Annotators	Tweets, n	Agreement, n (%)	IAA^a^
1	AN1+AU1	15,405	13,560 (88.02)	0.815
2	AN1+AN2	8016	6414 (80.02)	0.681
3	AU1+AN2	6906	6709 (97.15)	0.953
4	AN1+AN2+AU1	6906	—^c^	0.904^b^

^a^Interannotator agreement.

^b^Fleiss Kappa.

^c^Not applicable.

### Automatic Classification

Table 3 presents the results of the classification experiments, showing the F_1_ scores per class, the overall accuracy, and 95% CIs for the accuracy. The RF and SVM classifiers particularly show promising performances, without any feature engineering or parameter tuning. The performance on the abuse class is particularly lower, as expected, because of the low number of instances belonging to this class.

**Table 3:** Class-specific F1 scores, overall accuracy, and 95% CIs for the accuracy for 4 classifiers.

Classifier	Abuse	Consumption	Mention	Unrelated	Correct predictions and accuracy (N=3271), n (%)	95% CI
NB^a^	0.51	0.66	0.77	0.81	2257 (69.00)	67.4-70.6
SVM^b^	0.53	0.67	0.82	0.78	2388 (73.00)	71.4-74.5
RF^c^	0.30	0.66	0.81	0.79	2352 (71.90)	70.3-73.4
dCNN^d^	0.35	0.64	0.79	0.16	2355 (72.00)	70.3-73.5

^a^NB: naive Bayes.

^b^SVM: support vector machine.

^c^RF: random forest.

^d^dCNN: deep convolutional neural network.

## Discussion

### Tweet Contents and Sources of Disagreements

The iterative process undertaken for our guideline development was crucial to concretize the definitions for each of the classes and identify sample tweets presenting a multiplicity of types of information for each class, and to reduce decision-making uncertainties among the annotators. Through the process, we raised IAA from 0.569 in the first round to a combined average of 0.861, which can be interpreted as an “almost perfect agreement” [34]. Though we were able to increase overall agreement with improvements to the guidelines, the short and context-lacking nature of many tweets makes it hard to eliminate disagreements entirely. There are many tweets that do not unambiguously meet the requirements stated as identifying markers so that they can be definitively categorized, and the annotators must rely on their background knowledge and judgment. Table 4 shows several examples of difficult-to-categorize tweets and the eventual category assigned following disagreement resolution, along with justification for it. A more detailed listing of these examples is provided in the full guidelines (Multimedia Appendix 1).

**Table 4:** Examples of difficult-to-annotate instances.

Tweet	Category	Justification
generic xanax and adderall look way too alike. oh no what have i done...?	C^a^	There is inexplicit evidence that the user took the medication, although there is no evidence of abuse.
Going by a restaurant before 10:30 and not stopping to get breakfast is how you know you're on Vyvanse	C	There is inexplicit evidence that the user took the medication, although there is no evidence of abuse.
if this tweet sticks i'll eat my shorts (made of adderall)	A^b^	The user is expressing an intent to abuse, with an inexplicit indication that he/she has access to the medication.
i always freak out before a speech, always... this is the part where i'm supposed to ask my gp for zoloft or roofies but nooo,	M^c^	The user is expressing that he/she does not have access to the medication and expressing a situation.
i swear vyvanse got you finishing things you didn't know you had to doo #justironedmysocks	C	The tweet expresses the effect of Vyvanse more like a side effect, with no evidence or hint to indicate that the drug is being abused.
so glad i did my research and never let anyone convince me to take tysabri or gilenya. dr. was so informative!	M	The user is expressing that he or she never took the medication.
vyvanse i love you so much omg like i want to marry you i want to love you	C	The user is expressing love for Vyvanse, although never really expressing or hinting at possible abuse. If there was any hint of abuse, this tweet would be labeled as such.
took double dose vyvanse today by accident. i'mbouncinall around.	A	Although the misuse is unintentional, the user is expressing certain sensations brought about by the drug, so it was considered to be abuse-indicating. This is another borderline case.

^a^C: Non-abuse consumption.

^b^A: Potential abuse or misuse.

^c^M: Drug mention only.

We also performed a word-level analysis to better understand how the contents of the tweets belonging to the 4 classes differed, if at all. We found that the consumption tweets contain more health-related terms (eg, pain, anxiety, sleep, and doctor), whereas the unrelated tweets contain mostly irrelevant terms (eg, song, Anderson, and Hollywood). There are similarities in the word frequencies in the abuse or misuse and mention categories, indicating that discussion about abusing medications is not remarkably different from general discussions about the medications. This adds to the difficulty of accurately classifying the tweets belonging to the smaller abuse or misuse class.

In addition to the word-level similarities between the abuse or misuse and mention classes, the ambiguity in the language and the lack of context within the tweets leave them open to subjective interpretation, which affects the annotation process itself. These interpretations are troublesome when there can be multiple meanings in the clues that are present. For example, a tweet may have no explicit mention of abuse, but the use of certain keywords (eg, popped) or the situation may suggest that there might be misuse or abuse involved (possible abuse). However, it is not unreasonable that the use of such expressions would also be adopted by a patient taking their medication in the prescribed manner, making it difficult for the annotators to decide when it should be considered abuse and when it should be considered consumption. We sought to mitigate the effect of this uncertainty on the quality of the corpus by double, or even triple, annotating each tweet to achieve consensus.

#### Utility of Annotation Guideline and Data

The key objective behind creating detailed annotation guidelines and making them publicly available is to ensure the reproducibility of the annotation experiments. This is of particular importance for health-related data, from public social media or other sources such as electronic health records, which may have restrictions on public sharing, requiring researchers from different institutions to annotate their own data. For example, Twitter requires researchers to make a *reasonable effort* to remove data that are no longer in the public sphere. Therefore, data used in the training and testing of corpora may not be available as time passes, as users may delete tweets or change the privacy settings of their profiles. For a task such as the one we address here, new data may need to be collected and annotated in the future by other researchers (eg, to have comparable training data or to have a sample size with enough power to effectively train a machine learning classifier). The same is true if tweets mentioning medications not included in our sample are to be annotated for the same purpose in the future. Having a thorough, standardized annotation guideline may guide future annotation efforts. Furthermore, making the guidelines generalizable to the task rather than the data allows the methods to be transferred to other sources of similar social media data, such as Reddit or Facebook, so comparisons can be made about the utility of each source.

The expansion of the classes did decrease the accuracy we achieved from our prior pilot study [26] in which we modeled the problem as a binary classification one and had obtained lower IAA. The higher IAA raises the performance ceiling for supervised classification systems on these data. We have presented a set of automatic classification experiments and results, and, interestingly, the SVM classifier outperforms the dCNN classifier. The deep learning system particularly underperforms on the classes with few instances, which is a phenomenon we have observed in past classification tasks. The optimal classification strategy for such social media–based datasets is typically discovered via community-driven efforts such as shared tasks [12], and our objective is to enable that with this dataset. The identification of prescribed consumers of the medications may allow us to identify those users who later exhibit signs of abusing or misusing the medication and also potentially study long-term effects of such behavior. We leave these tasks as future work. The identification of mentions only and unrelated tweets will allow us to develop better filtering methods to ensure that a higher quality corpus is used for data collection and analysis, thus reducing the potential for biases and misleading conclusions [35].

#### Principal Findings

The principal findings and outcomes of the work described in this paper are summarized as follows:

1. Creation of annotated data that will be used to promote community-driven research focusing on social media mining for prescription medication abuse research. We have made the manually labeled training data available with this manuscript, and the evaluation set will be used to evaluate systems via shared tasks [12].
2. We have provided elaborate descriptions about how prescription medication misuse or abuse is discussed on Twitter for a number of medications. Our detailed annotation guideline may be used by others to contribute more annotated datasets involving additional sets of medications.
3. The machine learning results mentioned in the paper present strong baseline and benchmark results for future systems trained and evaluated on this dataset.

#### Comparison With Prior Work

A number of recent studies, including our preliminary studies on the topic [18,26,36], have explored the possibility of using social media for monitoring prescription medication abuse and have validated that it can serve as a potentially useful resource. Studies have suggested that reports of prescription medication misuse, including the use of specific formulations, temporal trends of abuse, and geolocation-based trends can potentially be discovered from social media—information that is not available from other sources because these are not voluntarily reported to health practitioners or agencies. Early studies have primarily attempted manual methods for qualitatively and quantitatively verifying the presence of abuse-related data from social media [18,37]. Later efforts attempted to automate the process of detection via NLP and machine learning approaches, or explore other aspects related to misuse (eg, user sentiments) [26,38]. Although there is consensus regarding the presence of valuable information in social media data, there is a lack of consistent methodologies for mining the information. Unsupervised approaches, for example, are suitable for analyzing data snapshots but not portable across time periods because of the evolving nature of the social media sphere. Due to the need for large training sets and the time and expense related to manually creating these datasets, weak supervision approaches have been explored as a means to create larger, albeit noisier, training data. However, these approaches may still require some labeled data or domain expertise to generate the data programming or feature labels [39,40]. The training data generated by these approaches may degenerate the performance over baseline approaches using only labeled data [41]. There is also a lack of publicly available annotated data that can be readily used by health informatics researchers to develop data-centric systems, or annotation standards using which consistent datasets can be built across institutions. Although supervised classification approaches have been shown to be promising for automatic detection of prescription medication abuse–related posts, the performances reported by systems are typically low for this task even when compared with other social media–based text classification tasks [26,42,43]. A contributing factor to these relatively low performances is the IAA rates that are typically low. For example, 2 recent papers reported IAA rates ranging from 0.45 to 0.46 for manual annotation [44,45] but no follow-up work to better define the annotation task or guidelines to improve the rates. We believe that the root of the problem of low agreement rates for this task is the lack of understanding or agreement regarding how users express medication abuse, or what constitutes misuse vs medical use. This problem does not exist, for example, in the task of illicit drug abuse annotation, in which any consumption can be regarded as abuse. In the case of prescription medications, it has to be determined if the drug is being consumed, and, if yes, if there is evidence of nonmedical consumption. The issue of such low agreement rates must be addressed for laying the foundations of long-term research on this topic and before releasing datasets for community-driven development of solutions. We attempt to address this as the primary focus of this paper by elaborately describing the chatter on Twitter, discussing annotation decisions and a guideline, and illustrating the utility of the developed corpus by presenting the results of several machine learning experiments.

#### Limitations

The study has several limitations, particularly in terms of scope. Only Twitter data are included in this study and the accompanying dataset, although data on misuse or abuse are also available from other social networks such as Instagram and Reddit [46,47]. We have included 20 abuse-prone medications although in reality there are other medications and categories of medications that are also prone to misuse or abuse. In addition, our study did not include illicit drugs, which is another branch of social media–based drug abuse research that has received considerable attention over recent years. We included medication names (generic and trade) and their common misspellings, but we did not use any street names for data collection. Future research may focus on including more illicit medication and establish annotation guidelines relevant for them, similar to our work presented here. We also included only the tweets that were in the English language, which limits the use of these data for training systems for English text only. However, our guidelines may be followed by future researchers to create annotated datasets in other languages. From the perspective of demographic representation, social media users are different from the actual population, with a larger representation of young people than older people.

#### Conclusions

In this paper, we discussed how users present information about prescription medication abuse and consumption on Twitter, described the iterative annotation of a large corpus containing 16,443 tweets, outlined our annotation guidelines that we have made available along with this publication, and presented the performance of several baseline classifiers over a sample of the corpus to demonstrate its utility. In our annotation guideline, we identified and defined 4 possible broad categories of topics of discussion related to abuse-prone prescription medications: potential abuse or misuse, non-abuse consumption, mention only, and unrelated. The guidelines were improved over a series of iterations of annotation and reviewed until we reached an agreeable level of consistency in our annotations. Through this process, we created a high-quality annotated corpus that can serve as the standardized dataset for future research on the topic. We expect that our annotation strategy, guidelines, and dataset will provide a significant boost to community-driven data-centric approaches for the task of monitoring prescription medication misuse or abuse monitoring from Twitter. Considering the growing problem of drug abuse, social media–based research may provide important unprecedented insights about the problem and perhaps even enable the discovery of novel abuse-prone medications or medication combinations.

## Appendix

Multimedia Appendix 1 Full annotation guidelines.

## Abbreviations

dCNN: deep convolutional neural network
IAA: interannotator agreement
NB: naive Bayes
NIDA: National Institute on Drug Abuse
NIH: National Institutes of Health
NLP: natural language processing
RF: random forest
SVM: support vector machine
