# Supplementary material for: Promoting Reproducible Research for Characterizing Nonmedical Use of Medications Through Data Annotation: Description of a Twitter Corpus and Guidelines
Source: J Med Internet Res. 2020 Feb 26;22(2):e15861. doi: 10.2196/15861 (PMC7066507; doi:10.2196/15861)
Supplement: Multimedia Appendix 1 [file jmir_v22i2e15861_app1.docx]

# **Toxicovigilance from social media: Annotation guidelines**

## **Contributors:**

Karen O’Connor^1^

Annika DeRoos^2^

Alexis Upshur^1^

Graciela Gonzalez-Hernandez^1^

Jeanmarie Perrone^3^

## Abeed Sarker^4^

1. Department of Biostatistics, Epidemiology and Informatics, Perelman School of Medicine, University of Pennsylvania
2. College of Arts and Sciences, University of Pennsylvania
3. Department of Emergency Medicine, Perelman School of Medicine, University of Pennsylvania
4. Department of Biomedical Informatics, School of Medicine, Emory University

Version: 3.0

Last updated: October 20, 2019

**Purpose**

The goal of the annotation task is to label tweets as one of three categories: “*drug mention only*” (no evidence of abuse or consumption), “*abuse-indicating*” (tweets that indicate past abuse or intent to abuse), “*non-abuse/consumption*” (tweets that mention the medication name in the context of personal consumption/intake but do not indicate any form of abuse or misuse) and “*unrelated*” (tweets which contain the drug name as keyword, but are referring to something else). The following guidelines will help annotators, with examples, to determine which tweets should be considered as *abuse-indicating*.

**Introduction**

Our annotation will classify the tweets into three categories:

1. *Potential Abuse/Misuse* (A): These tweets contain possible indications that the users is abusing or is seeking to abuse or misuse the medication. The user may have a valid prescription for the medication but their manner of use is indicative of abuse/misuse, or the medication may have been obtained illegally. In our annotation, we also include in the *abuse/misuse* class tweets that can possibly indicate abuse without confirming evidence. Timelines of users who ‘*possibly*’ mention abuse need also be studied, so such tweets should be labeled as misuse/abuse. This category also includes report of misuse by an immediate family member (*e.g.*, brother, mother etc.) or abuse by a third person who is related to the user.
2. *Non-abuse/Consumption* (C): These tweets indicate that the user has a valid prescription for the medication and is taking the medication as prescribed; or is seeking to obtain the medication for a valid indicated reason. Tweets should be categorized as this when there is evidence of possible consumption but there is no evidence of abuse/misuse. This category only applies to personal consumption.
3. *Drug Mention Only* (M): In these tweets the mention of the medication name is not related to wanting, needing, or using the medication either as prescribed or misuse/abuse. These tweets may be sharing information or news about the medication, jokes, movie or book titles, lines from movies or songs, etc.
4. *Unrelated* (U): These tweets mention the medication/drug names, but they don’t represent the drug and refer to something else.

We now provide some examples of annotations along with explanations in order to help annotators with their decision-making.

**1. Identifying Abuse-indicating Tweets (A)**

According to the FDA^[[1]](#footnote-1)^ the following definitions are provided for *misuse* and *abuse*:

When a person takes a legal prescription medication for a purpose other than the reason it was prescribed, or when that person takes a drug not prescribed to him or her, that is misuse of a drug. Misuse can include taking a drug in a manner or at a dose that was not recommended by a health care professional. This can happen when the person hopes to get a bigger or faster therapeutic response from medications such as sleeping or weight loss pills. It can also happen when the person wants to “get high,” which is an example of prescription drug abuse.

If a person knows that they will get a pleasant or euphoric feeling by taking a drug, especially at higher doses than prescribed. That is an example of drug abuse because the person is specifically looking for that euphoric response. In contrast, if a person isn’t able to fall asleep after taking a single sleeping pill, they may take another pill an hour later, thinking, “That will do the job.” Or a person may offer his headache medication to a friend who is in pain. Those are examples of drug misuse because, even though these people did not follow medical instructions, they were not looking to “get high” from the drugs.

Based on the end goals of this project, we do not differentiate between misuse and abuse.

(i) The tweet explicitly states that the user has taken or is going to take the medication to experience certain feelings (*i.e.*, to get high) or that the user experienced certain feelings in the past

- when you don't take a vyvanse for more than three weeks and then you do you **get high** as shit for a little
- man fuck the person who introduced me to fluoxetine and olanzapine ..who decided it was smart to fuck with crazy people drugs .. **#loopy. ^_^**
- maybe that second purple drink and liquid hydrocodone mix was overkill.... **i'm seeing ghost shit out the corner of my eyes down the hallway**
- @username @username i'm just taking some more codeine with my diazepam before i leave. **i feel epic!**

(ii) The tweet expresses that the user has or is going to take an increased amount of a medication

- you know you took **too much adderall** when you stalk your own facebook profile back to 2007
- gonna **double up on my prozac** tonight and hope to god things can only go up from here.
- when you take **too much adderall** and it gives you the jitters &lt;&lt;&lt;&lt;&lt;&lt;&lt;&lt;&lt;
- let's see how fast a **double dose** of hydrocodone will knock me out #thewaitinggame

(iii) The tweet expresses that the user has or is going to co-ingest a medication with other prescription medications or illicit drugs or alcohol or coffee (or other substances)

- time for my daily afternoon relaxation ritual of **smoking weed, taking 2 mgs of clonazepam, and 400 mg of seroquel xr**.
- why just **drink a lot or just take a seroquel to get to sleep when you can do both**?
- never forget the 2 most important a's of college: **alcohol and adderall** 🍻💊
- **adderall and red bull** work magic @username and @username #round2

(iv) The tweet expresses a mechanism of intake that is typically associated with abuse/misuse

- alright so the plan is. **snort** adderall then count the stars in a star wars movie
- **snorted** a line of oxycodone last night &amp; swear i was feeling alllll types of waysss since i was on molly.
- @username one time i **shoved adderall up my ass** and since your intestines are super absorbant i got jacked as fuck
- shot some of that oxy **thru ma veins**

(v) The tweet suggests that the user has access to prescription medications and/or illicit substances, and there is a hint in the tweet that the user may be involved in misusing/abusing the medication.

- i may not have weed, but **i do have 5 seroquel** i found while cleaning my room :3 -g
- i **found an adderall in my carpet** today and sat at my computer on adobe illustrator for hours and hours just making shit
- had a dream that i found some **adderall** to help me stay awake at work and when i woke up **i did find some**
- i was really exhausted at work then i **found a random 30 adderall** in my purse there is a god

(vi) The tweet describes the user giving the medication to a third person for abuse/misuse (who is not prescribed the medication) and/or it describes that the user is abusing/misusing the medication with a third person or a group of people.

- im bout to slip some of my **seroquel into her drink**. she gonna be knocked
- **my roommate took one of my vyvanse** and has pooped like 4 times. confirmation: girls poop.
- @username thanks gail, havin a lil **help from my friends oxycodone** &amp; cannacap. (i come back friday) ??????

(vii) The tweet discusses abuse-prone prescription medication and/or illicit drug trading by themselves or by an acquaintance (not just general statements about drug trading).

- literally tho, could someone **sell me an adderall or vyvanse**? i just got paid
- anyone **want to buy valium or adderall** because i have an abundance and don't fuck with either
- shout out to the guy at savers who **sold me vyvanse (shout out to me for buying it)** and got me hooked on adhd meds since
- goin' to the x-games, got a suitcase full of adderall and dexedrine time to make some cash **selling it to cod teams #esports #dare**

(viii) The tweet suggests that the user takes or will take a prescription medication for performance enhancement or in order to complete a task. Note that in many cases, the user might actually be prescribed a medication which he/she needs to take to complete a task. So, the annotator has to look for hints of abuse, indicating that the user is misusing/abusing the medication

- and now is when we pray to the adderall gods for **solid learning enhancement**.
- i have **so much to do and not a lot of time to do it**. adderall to the rescue, i guess.
- Time for some vyvanse and **finish this assignment tonight**!

(ix) The tweet suggests that the user has taken or will take a medication for a purpose or desired long-term physical effect (*e.g.*, weight loss) that is not what the medication is for

- fuck working out, i'm done with that shit, it's been a month and i've gained weight. **back to vyvanse and copious amounts of iced coffee**.
- if u **wna lose weight just do what i did -start smoking cigarettes +get a vyvanse prescription!** youll wna die but atleast ur ribs will show:)

(x) There is no explicit mention of abuse, but the use of certain keywords (*e.g.*, popped) or the situation suggest that there might be abuse involved (possible abuse). The posts belonging to this subcategory might be vague and so the annotator has to use judgment to decide when it should be considered abuse and when it should be considered intake.

- i **took a left over vyvanse** i had from finals week so in about an hour if i start tweeting a lot i'm sorry
- mom: how many **xanax pills** did you take? me: **good vibes and good times mom chill**.
- chinese an cake jus do wat i say an nobody die **i open tha soda i get tha codeine an i poured up a lot**
- i found a **random bag of adderall in my room earlier today** and then lost it somewhere else in my house ???? #freakingout #cantfindit
- line of adderall at 11:05? no thanks. i'll stick to my ganja and make fun of you till you fall asleep at 4am. #weedwins #adderallloses

(xi) The tweet suggests that the user is addicted on a medication or is having adverse effects due to discontinuation of the medication in non-standard doses

- i convinced my mom to give me a xanax at 7 for my panic attacks but i just realized i need to save it for the comedown. **i'm craving it now**
- here's where **my addiction to codeine** began. i bought codeine over the counter for the constant pain i had in my left side.
- could just be **lethargy from taking 15 doses of synthroid** when i panickly realised i stopped taking it to avoid getting fat

(xi) Possible category (need to discuss this): abuse by a family member (there are studies suggesting that once a family member is abusing, other family members are also more likely to do it) or a close friend. If a tweet mentions a third person with a clear indication of a close relationship with the user and the tweet also mentions abuse/misuse, it should fall under this category. Generic third person descriptions of abuse/misuse without an indication of closeness with the user should not be categorized as abuse. (For further clarification, see the examples in point (vi) in 3. Identifying Mention Tweets).

- hey i know you were fucked at the head at one point so can you help me out- **my cousin asking for adderall**

**2. Identifying Non Abuse Indicating Tweets Representing Personal Consumption (C)**

This category should include tweets from which it can be derived that the user has taken/consumed the medication in the past or is going to take it, but there is no evidence of abuse.

1. The tweet mentions the condition treated by the medication which matches its indicated use, and nothing in the tweet implies that they are misusing/abusing the medication
   - #knowyouranon **ive taken zoloft (antidepressants) and vyvanse (adhd meds) for over a year now**
   - @username that may be why it reduces anxiety and depression in some and can help with ptsd symptoms. **i use it for pmdd**, 40mg prozac
   - **i was recommended vyvanse** by my doctor to help me focus. i took my first one today and i feel more jittery than focused
   - plus-side of my life, **enbrel is working, psoriasis is leaving**! maybe my mental state will continue to improve as well. ...lol...
   - i'm on like **80mg of prozac and trazedome and im still crazy and bipolar seeming**
   - @username **adderall** b like "hey don't you have normal life to deal with too late you are organizing your sock drawer for 2hr" #**adhdlife**
2. The user mentions side effects of the drug, but there is no implication that these are the result of misusing/abusing the drug
   - **prozac made me lose my mind**, see things and have increased suicide thoughts. but the government calls it safe.
   - **still effexor withdrawal**. this is the last time i ever take anti depressants.
   - **lamictal is nice because it makes me chill** out but it **also makes me feel like i'm gonna throw up everywhere**
   - vyvanse is the devil. **why am i still awake?! why have i only slept for one hour?** why is it not out of my system by now? 😳😁👎
3. In the tweet, the user expresses a want for the medication for a condition that matches its indicated use
   - i must seriously **suffer from that sad need more sunshine** please! thanks in advance or do i need to order the **prozac** xx #bleurghh
   - interested in the **pain relief aspect of cymbalta** but not the horrific withdrawal if i ever go off of it (ssri withdrawal is bad enough).
   - has anyone taken **pregabalin for anxiety**? my old psych suggested it (before the **new ones put me on quetiapine**) &amp; thinking of trying to &gt;
4. The user talks about personal experience from the past or makes inexplicit suggestions that he/she is prescribed the medication or has been taking it, without any further evidence of abuse

- if you ever need to clean your room, take a vyvanse and then you'll **clean your room, your car and the rest of the house. and the yard**.
- **there isn't enough vyvanse in the world to get me through** a traffic jam without totally losing my shit.
- **adderall** has me having 15 minute conversations about piercings with the cashier at publix

1. The tweet indicated the user has a prescription for the medication, or has been on the medication for an extended period of time indicating that the medication is prescribed
   - **my doc says vyvanse** is supposed to last 12 hours. uhhhhhhh. is that true for any of you? def not true for me
   - actually don't feel ready to come off **my fluoxetine** anymore, asdfghjkl i just dont know
   - **doc increased my add med &amp; prozac dose**. hoping for improved focus, ability to stay awake all day. that mixed with workout plan should help!
   - forgot to take **my vyvanse** this morning so i might as well not even be at school
   - i had to be taken of nuvigil today because of a reaction i was having, **doctor will likely try adderall next** .. what meds have worked for you
   - **got switched to vyvanse and off concerta** so probably going to loose like 10 pounds
   - i have a **prescription for paxil** now. thanks to everybody that was real w/ me about depression stuff
   - @username i have ms too. i have been **on tysabri for 4 years**, and diagnosed for 11. i'm 26, and doing well, what r u doing for treatment?
   - where is my **mother to force my adderall down my throat** &amp; make me do 2849392883 hours of calc/accounting....mother?!?!

**3. Identifying Drug Mention Only Tweets (M)**

1. The tweet conveys some information about the medication but contains no indication that the user is taking or wants to take the medication
   - fda recommends **paroxetine (paxil)** not be prescribed for children. risk of suicide and lack of efficacy #teenhealth
   - **fosamax** has been linked to many side effects that include femur fractures, esophagus problems &amp; hardening of the jaw. 1.888.520.5202.
   - ok just saw a commercial for a medicine called **humira** for crohn's disease but i'm 99% sure a week ago it was for a different disease. not ok
   - **ciprofloxacin** is active against both gram-positive and gram-negative bacteria. it is particularly active against gram-negative bacteria.....
   - the **adderall** tweets are not even funny to me. if you saw what i see at work daily it wouldn't be funny to you either. #sosad
2. The mention of the medication is from a song, book or movie, or some other cultural reference
   - @username a clockwork orange is super good if you like dystopias or **prozac nation** which is really depressing but one of my favorites. (**book title**)
   - @username narly, radical, on the block i'm magical. see me at your college campus, baggie full of adderall 👅 (**song lyric**)
   - adderall to stay focused xanax to take the edge off pot to mellow me out cocaine to wake me back up again and morphine..well b/c its awesome (**quote from a movie**)
   - @username a big improvement after going to seahawks? that adderall may have helped **(scandal involving seahawk players used PEDs including adderall**)
3. The mention of the medication is being used in a joking or a hypothetical statement
   - we used to take acid to feel different, nowadays we take **prozac** to feel normal
   - this substitute teacher must have taken an **adderall** just to teach the class, she's jittery af
   - anyone else feel like professors should go easier on people who don't have an **adderall** prescription..,..
   - she tweeting like she's on **adderall** 😂😂😭@username @username @username #r5mcplaytakeover <http://t.co/c31kwfsnug>
   - i can't stop laughing about the @username skit about second-term strength **paxil** - not covered by obamacare.
4. Any other general mention of the medication where the is no indication in the tweet that the user is taking or wants to take the medication
   - seeing a boatload of **duloxetine** here the past several days, too. @username
   - gary lewis just mentioned we as students take **prozac** during finals......whut....
   - morgans dog name is addi and this drunk guy keeps calling her **adderall** 😭
5. Other people’s usage with no indication of closeness (*e.g.*, family, close friend etc.) to the user or evidence that the user is involved in the use/abuse
   - one of my favorite kids is indigo and **his mom gives him adderall** :( i told her to stop that shit
   - **megan has to take an adderall** so she can be more chatty and be able to talk to boys lol
   - when finals rolls around i can't tell if people are really social and nice to me because that's just who they are **or if they are on adderall**
   - if **my group member shows up high again tomorrow it better be from adderall** and not weed because nobody got time for a stoned group member
   - “**@username: trying to sleep.. but this vyvanse is keeping me up**” rmft
   - only @username would **ask her dad if she should take an adderall** tomorrow 😂😂

(vii) Retweets and general statements

- - 85% of a university student's diet at this time of year consists of coffee and **adderall** #unilife #statscan
  - RT don't pop e, **adderall** &amp; drink straight liquor in one night then decide to have sex. you will wake up 3 weeks later pregnant. be smart lmao

**4. Identifying Unrelated Tweets (U)**

(i) The only tweets that belong to this category are those that include a drug/medication name as keyword, but the keyword is referring to something else and not the drug/medication. It can be, for example, a person’s name or a misspelling of something else

- - Nothing cleans these clothes like **oxy** does.. and the smell too!
  - @username @username 6 books AND a master's thesis? OK, you are now officially my heroine.
  - keyla said she didn't want to sleep with drea , because the dog sinks so , now she's in here **with me and lyrica**
  - (#ig_haiiddy_x13) **lyrica anderson** ft wiz khalifa – freakin: lyrica anderson ft wiz khalifa – freakin download: lyri... (#ig_haiiddy_x13)
  - “@username: cannot believe **what savella just said** to me looool” / loool mikro joke orrr? 😂
  - how are people calmly tweetig **song lyrica** we are 20k bhind #votekatniss

**Questions that arose and Examples of difficult instances**

**Questions:**

Q: What to do if the tweet is mostly in a foreign language?

A: We should not try to interpret from a foreign language and decide based on the English part. If we can't infer that they are talking about the medication (i.e., most of the tweet is non-English), we should label as unrelated.

Q: How to we label tweets that do not make sense (word salad tweets), or those that don’t give enough to really infer anything?

A: If the word salad contains other drugs or usual co-ingested substances, we mark as potential abuse. Otherwise as mention.

Q: In the guidelines, tweets with a co-ingested substance, such as coffee, are classified as ‘potential abuse’ but some mentions don’t seem to be suggesting any abuse-related behavior, should they still be labeled abuse?

A: If it appears that they are taking coffee in some form of misuse of the medication, then yes it should be abuse. Otherwise, we can leave as consumption or mention, depending on the context.

**Examples of difficult instances:**

| **Tweet** | **Category** | **Justification** |
| --- | --- | --- |
| generic xanax and adderall look far too similar. oh no what have i done...? | C | There is inexplicit evidence that the user took the medication, although there is no evidence of abuse. |
| passing a chickfila before 10:30 and not stopping to get breakfast is how you know you're on vyvanse | C | There is inexplicit evidence that the user took the medication, although there is no evidence of abuse. |
| @username if this tweet sticks i'll eat my hat (made of adderall ) | A | The user is expressing an intent to abuse, with an inexplicit indication that he/she has access to the medication. |
| i always panic before a paper, always... this is the part where i'm supposed to ask my gp for prozac or roofies but nooo, @username | M | The user is expressing that he/she doesn’t have access to the medication and expressing a situation. |
| i swear vyvanse got you finishing things you didn't know you had to doo #justironedmysocks | C | The tweet expresses the effect of vyvanse more like a side effect. No evidence or hint that the drug is being abused. |
| so glad i did my research and never let no one to convince me to go on tysabri or gilenya. dr. @username was so informative! | M | The user is expressing that he/she never took the medication. |
| vyvanse i love you so effin much omg like i want to marry you i want to comfort you i want to make love to you i want to love you baby mmmmm | C | The user is expressing love for vyvanse, although never really expressing or hinting at possible abuse. If there was any hint of abuse, this tweet would be labeled as such. |
| @username took double dose vyvanse today by accident. i'm bouncin of da wallz. | A | Although the misuse is unintentional, the user is expressing certain sensations brought about by the drug, so it was considered to be abuse-indicating. This is another borderline case. |

**Table 1.** Examples of difficult to annotate instances.

**Funding:**

Research reported in this publication was supported by the National Institute on Drug Abuse of the National Institutes of Health under Award Number R01DA046619. The content is solely the responsibility of the authors and does not necessarily represent the official views of the National Institutes of Health.

1. <https://www.fda.gov/ForConsumers/ConsumerUpdates/ucm220112.htm>. Accessed 5/4/18. [↑](#footnote-ref-1)
